# Supplementary material for: Impact of SARS-CoV-2 Infection on Unvaccinated Pregnant Women: Non-Reassuring Fetal Heart Rate Tracing Because of Placentitis
Source: Viruses. 2023 Apr 27;15(5):1069. doi: 10.3390/v15051069 (PMC10223018; doi:10.3390/v15051069)
Supplement: Supplementary file 1 [file viruses-15-01069-s001.zip › viruses-2247520-supplementary.pdf]

Table S1. NIH classification of SARS-CoV-2 clinical severity [16].

Asymptomatic or presymptomatic infection	Individuals who test positive for SARS-CoV-2 using a virologic test (i.e., a nucleic acid amplification test [NAAT] or an antigen test) but who have no symptoms that are consistent with COVID-19
Mild illness	Individuals who have any of the various signs and symptoms of COVID-19 (e.g., fever, cough, sore throat, malaise, headache, muscle pain, nausea, vomiting, diarrhea, loss of taste and smell) but who do not have shortness of breath, dyspnea, or abnormal chest imaging.
Moderate illness	Individuals who show evidence of lower respiratory disease during clinical assessment or imaging and who have an oxygen saturation (SpO_2) ≥94% on room air at sea level.
Severe illness	Individuals who have SpO_2 <94% on room air at sea level, a ratio of arterial partial pressure of oxygen to fraction of inspired oxygen (PaO_2/FiO_2) <300 mm Hg, a respiratory rate >30 breaths/min, or lung infiltrates >50%.
Critical illness	Individuals who have respiratory failure, septic shock, and/or multiple organ dysfunction.

Table S2. Maternal biochemical characteristics.

**Maternal biochemical characteristics**	
Hemoglobin (*n* = 17)	
Diagnosis (*n* = 13), g/dL	11.7 (10.8–12.7)
Hospitalization, g/dL	12.1 (10.2–12.9)
Delivery, g/dL	12.1 (10.1–12.9)
Platelet count (*n* = 17)	
Diagnosis (*n* =14), G/L	153 (120–216)
Hospitalization, G/L	136 (106–218)
Delivery, G/L	122 (62–218)
Thrombocytopenia < 100 G/L<br>- Diagnosis<br>- Hospitalization<br>- Delivery	2 (14.3)<br>4 (23.5)<br>7 (41)
Lymphocyte count (*n* = 16)	
Diagnosis (*n* = 13), ×10^3/mm^3	0.8 (0.5–0.9)
Hospitalization, ×10^3/mm^3	0.7 (0.5–1.3)
Delivery, ×10^3/mm^3	1.3 (0.8–1.7)
Lymphocytopenia < ×10^3/mm^3 (no, %)<br>- Diagnosis<br>- Hospitalization<br>- Delivery	10 (71.4)<br>10 (62.5)<br>6 (37.5)
APTT ratio (*n* = 16)	
Diagnosis (*n* = 10)	1.2 (1.1–1.4)
Hospitalization	1.4 (1.2–1.7)
Delivery	1.3 (1.1–1.6)
Prolonged APTT > 1.2<br>- Diagnosis<br>- Hospitalization<br>- Delivery	6 (60)<br>11 (68.7)<br>10 (62.5)
SGOT (*n* = 12)	
Diagnosis (*n* = 7), U/L	37 (28–76)

Hospitalization, U/L	70 (50–94)
Delivery, U/L	85 (46–108)
SGOT > 2 N<br>- Diagnosis<br>- Hospitalization<br>- Delivery	3 (42.8)<br>5 (41.6)<br>7 (58.3)
SGPT (*n* = 12)	
Diagnosis (*n* = 7), U/L	13 (8, 45)
Hospitalization, U/L	34 (20, 48)
Birth, U/L	64 (20, 90)
SGPT > 2 N<br>- Diagnosis<br>- Hospitalization<br>- Delivery	1 (14.2)<br>2 (16.6)<br>7 (58.3)
Fibrinogen (*n* = 13)	
Diagnosis, g/L	4.3 (2.3–5.1)
Hospitalization, g/L	3.4 (1.2–5.1)
Birth, g/L	2.8 (1.2–4.3)
Fibrinogen < 1.5 g/L<br>- Diagnosis<br>- Hospitalization<br>- Delivery	1 (8)<br>4 (30)<br>5 (38)

Median (IQR) or *n* (%) are provided. APTT: activated partial thromboplastin time. SGOT: serum glutamic-oxaloacetic transaminase. SGPT: serum glutamic-pyruvic transaminase.
